# A post-irradiation-induced replication stress promotes *RET* proto-oncogene breakage

**DOI:** 10.1530/ETJ-24-0028

**Published:** 2024-08-23

**Authors:** Fabio Hecht, Laura Valerio, Carlos Frederico Lima Gonçalves, Marylin Harinquet, Rabii Ameziane El Hassani, Denise P Carvalho, Stephane Koundrioukoff, Jean-Charles Cadoret, Corinne Dupuy

**Affiliations:** 1 Université Paris-Saclay, Orsay, France; 2 UMR 9019 CNRS F-94805 Villejuif, France; 3 Gustave Roussy, Villejuif, France; 4 Laboratoire de Biologie des Pathologies Humaines ‘BioPatH’, Université Mohammed V de Rabat, Faculté des Sciences, BP1014 Rabat, 10001, Morocco; 5 Laboratório de Fisiologia Endócrina Doris Rosenthal, nstituto de Biofísica Carlos Chagas Filho, Universidade Federal do Rio de Janeiro, Rio de Janeiro, Brazil; 6 Sorbonne Université, Paris, France; 7 Université Paris Cité, CNRS, Institut Jacques Monod, Paris, France

**Keywords:** post-irradiation, replication stress, *RET*, thyroid cancer

## Abstract

**Objective:**

Ionizing radiation generates genomic instability by promoting the accumulation of chromosomal rearrangements. The oncogenic translocation *RET/PTC1* is present in more than 70% of radiation-induced thyroid cancers. Both *RET* and *CCDC6*, the genes implicated in *RET/PTC1*, are found within common fragile sites – chromosomal regions prone to DNA breakage during slight replication stress. Given that irradiated cells become more susceptible to genomic destabilization due to the accumulation of replication-stress-related double-strand breaks (DSBs), we explored whether *RET* and *CCDC6* exhibit DNA breakage under replicative stress several days post-irradiation of thyroid cells.

**Methods:**

We analyzed the dynamic of DNA replication in human thyroid epithelial cells (HThy-ori-3.1) 4 days post a 5-Gy exposure using molecular DNA combing. The DNA replication schedule was evaluated through replication-timing experiments. We implemented a ChIP-qPCR assay to determine whether the *RET* and *CCDC6* genes break following irradiation.

**Results:**

Our study indicates that replicative stress, occurring several days post-irradiation in thyroid cells, primarily causes DSBs in the *RET* gene. We discovered that both the *RET* and *CCDC6* genes undergo late replication in thyroid cells. However, only *RET*’s replication rate is notably delayed after irradiation.

**Conclusion:**

The findings suggest that post-irradiation in the *RET* gene causes a breakage in the replication fork, which could potentially invade another genomic area, including *CCDC6*. As a result, this could greatly contribute to the high prevalence of chromosomal *RET/PTC* rearrangements seen in patients exposed to external radiation.

## Introduction

Ionizing radiation (IR) leads to various delayed cellular effects, including chromosomal rearrangements, which are believed to play a key role in radiation-induced carcinogenesis. The thyroid gland is particularly sensitive to IR’s carcinogenic effects, whether from accidental or therapeutic exposure. The likelihood of thyroid tumors is at its highest when exposure happens at a young age, and the risk increases proportionally with the radiation dose (1). Over 90% of these tumors are papillary, with a *RET/PTC* chromosomal rearrangement found in 70% of cases (2).

*RET/PTC1*, the most prevalent type of *RET/PTC* rearrangement, is an intra-chromosomal paracentric inversion that results in a fusion between the 3′ portion of the *RET* gene (which encodes the receptor tyrosine kinase) and the 5′ part of the *CCDC6* gene (3). This leads to the production of a fusion protein with intrinsic tyrosine kinase activity, which prompts tumorigenesis in thyroid follicular cells. Both the *RET* and *CCDC6* genes are located within common fragile sites (CFSs) FRA10G and FRA10C, respectively (4).

CFSs are regions susceptible to DNA breakage under conditions of mild replication stress that impede DNA synthesis (5). They are considered hotspots for genomic instability and contribute to the development of cancer-specific chromosomal abnormalities. The application of fragile site-inducing chemicals, such as aphidicolin and bromodeoxyuridine (BrdU), can cause breakage in *RET* and *CCDC6*, respectively, demonstrating the specificity of fragile site induction (4).

DNA replication timing, which can vary across different tissues, also influences a cell’s vulnerability to replication stress (6). Late replication is a critical characteristic of several CFSs, as this can result in incomplete replication at the onset of mitosis, leading to DNA breakage (7).

In this study, we show that replicative stress materializes several days post-irradiation, triggering DNA double-strand breaks (DSBs), especially within the *RET* gene. Although both *RET* and *CCDC6* genes carry out late replication in thyroid cells, only the replication speed for *RET* is affected by irradiation. Collectively, our findings offer insights into why the *RET* gene is more prone to breakage after irradiation and why it often contributes to *RET* rearrangements in patients exposed to external radiation.

## Materials and methods

### Cell culture conditions

The human thyroid epithelial cell line (HThy-ori-3.1) was grown using the methods outlined previously (8). For testing, cells were grown in phenol red-free RPMI 1640 medium (Gibco) with a 5% FBS supplement. Uniform cell density and medium volume were used consistently throughout the experiment. Usually, 150,000 cells were seeded in 2 mL fresh medium across the wells of a six-well plate (9.62 cm^2^/well). This seeding density was selected to keep the cells proliferative until the experiment’s conclusion. Hence, this optimal density of 1.5 × 10^5^ cells/9.62 cm^2^ was referenced as the experiment’s optimized density. The human papillary thyroid carcinoma cell line (TPC-1) was grown in Dulbecco’s modified Eagle medium (Gibco) with GlutaMAX and high glucose supplemented with 10% FBS and 100 U/mL penicillin/streptomycin.

### X-ray irradiation

Cells were irradiated using an XRad320 X-ray generator (Precision X-Ray, Madison, CT, USA) 24 h after plating. The generator, operating at 320 KV/4 mA, delivered a dose rate of roughly 1 Gy/min. The samples were positioned 51.5 cm from the source and were exposed for 309 s, which equates to a 5 Gy dose. The culture flask media was replaced a few minutes before the irradiation and then again 24 h afterward, without further changes until the conclusion of the experiment.

### SDS-PAGE and Western blotting procedures

Western blot analysis was performed with lysates prepared as previously described (9). We probed the membranes with primary antibodies anti-γH2AX (Millipore, 05-636) and anti-H2AX (Abcam, Ab11175) overnight at 4°C with continuous agitation. Afterward, the membranes were washed three times with TBS-T and incubated for 45 min at room temperature with either goat anti-rabbit IgG-HRP antibody (Southern Biotech, 4010-05) or goat anti-mouse IgG-HRP antibody (Aglient Technologies, P0447). We then washed the membranes three more times with TBS-T and visualized the proteins using enhanced chemiluminescence.

### Cell cycle

Cells were seeded in six-well plates at an optimal density and then irradiated. Four days post-irradiation, cells were harvested using trypsinization, rinsed with PBS, and re-suspended in 500 μL of PBS. Next, 2 mL of cold 100% ethanol was gradually introduced to swirl and reach a final concentration of 80% before storing at −20°C for subsequent analysis. DNA detection was conducted by incubating the fixed cells for 30 min with a propidium iodide solution containing 1 mg/mL DNase-free RNase A (Sigma) and 0.4 mg/mL propidium iodide (Thermo Fisher Scientific) in PBS. The analysis was carried out using a BD Accuri TM C6.

### Replication-timing experiments

We used a protocol based on Hadjadj *et al*. (10), modified only slightly in the amplification step. DNA was amplified using Seq-plex, following the manufacturer’s instructions (Sigma). Sorted cell fractions were then labeled with either Cy3 or Cy5 ULS molecules, as recommended by Kreatech Biotechnology. The hybridization process adhered to the guidelines provided for 4x180K human microarrays (SurePrint G3 Human CGH Microarray Kit by Agillent Technologies, genome reference Hg18), which map the entire genome with one probe every 13 kb. Microarrays were scanned with Agilent’s High-resolution C scanner at a resolution of 3 μm, utilizing the autofocus feature. Results were analyzed through the START-R software (11), which generated replication-timing profiles.

### Immunofluorescence

Cells were seeded in six-well plates, each containing five circular coverslips. The process of irradiation and medium replacement was the same as described earlier. Four days post-irradiation, cells were fixed using warm 4% paraformaldehyde (Electron Microscopy Sciences) in PBS for 10 min.

We then washed the fixed cells thrice with PBS and permeabilized them using 0.1% PBS-Triton X-100 (Sigma) for 10 min. The cells were treated with a blocking buffer (PBS + 3% BSA) for 1 h to reduce non-specific binding. Next, we incubated the cells in a humid chamber at room temperature for 2 h with primary antibodies (anti-53BP1 and anti-cyclin D1) diluted in a blocking buffer.

After incubating, cells were washed thrice with PBS containing 0.1% Tween and exposed to secondary antibodies mixed with a Hoechst solution to counterstain the nuclei for 1 h. This was followed by three rounds of 5-min washes. The coverslips were then set in a fluorescent mounting medium (Faramount).

We observed the resulting immunofluorescence using an inverted microscope (Zeiss Axio Observer Z1) with an attached AxioCamMR3 camera at 20× magnification. We used ImageJ software for image processing and analysis.

A specific module was created for nuclear segmentation, based on DAPI signal intensity, to identify each nucleus separately. Focus segmentation for 53BP1 was facilitated by an integrated spot-detection module. All of the segmentation and pixel quantification values gathered from each cell/foci, including mean and total intensities, area, and number of foci, were exported to a custom software for further examination (12).

### Molecular DNA combing

We performed molecular DNA combing as detailed in references (6, 13). Initially, we plated cells at an optimized density in 6-cm Petri dishes and subjected them to irradiation. Four days post-irradiation, we labeled the neo-synthesized DNA with two successive 30-min pulses of iododeoxyuridine (IdU, 20 μM) and chlorodeoxyuridine (CldU, 100 μM) provided by MERCK. After that, we embedded the cells in a low-melting agarose block and purified the DNA prior to suspending it in 0.25 M MES with a pH level of 5.5. We then stretched the DNA fibers on silanized coverslips, ready for the immunodetection of IdU, CldU, and DNA counterstaining. We used a motorized stage-equipped Axio Imager.Z2 microscope from Zeiss to image the results, with scanning facilitated by the Metamorph software. Following this process, we measured 150 IdU-CldU tracks to calculate the replication speed in kb/min.

### ChIP-qPCR

Cells were plated in 175 cm^2^ flasks at the optimal density and irradiated after 24 h. Four days post-irradiation, they were trypsinized, washed, and counted. Approximately 10 × 10^6^ cells for each condition were cross-linked with 0.37% formaldehyde (SIGMA) for 10 min at room temperature with a rocking shaker. The formaldehyde was then quenched with 0.125 M glycine for 5 min. The cells underwent centrifugation at 800 ***g*** at 4°C for 5 min and were washed twice with PBS containing protease inhibitors (SIGMA). They were then re-suspended in hypotonic lysis buffer (containing 1 mM DTT, 15 mM MgCl_2_, 100 mM KCl, and 100 mM HEPES pH 7.9) with protease inhibitors and set on ice for 15 min. We then added 0.6% IGEPAL (SIGMA) and briefly vortexed the cells before centrifugation at 10,000 ***g*** for 30 s. We re-suspended the resulting nuclei in 2 mL of PBS and sonicated them using the Covaris® instrument for 10 min at 4°C to produce DNA fragments averaging 500 bp.

For each immunoprecipitation (IP), we diluted 30 μg of the fragmented DNA in IP dilution buffer and incubated it with 3 µg γH2AX antibody (Millipore, 05-636) or IgG isotype control (Abcam, ab81032), 20 µL of Magna ChIP beads (SIGMA), and protease inhibitor cocktail (SIGMA) overnight at 4°C with rotation. After magnetic separation, we discarded the supernatant and rinsed the beads with Low Salt Immune Complex Wash Buffer, High Salt Immune Complex Wash Buffer, and LiCl Immune Complex Wash Buffer, followed by another round of magnetic separation. The final wash was done using 1 mL TE buffer, and the beads were re-suspended in 100 µL TE buffer.

Subsequently, we deproteinized the samples through the addition of proteinase K (200 µg/mL) (Thermo Fisher Scientific) and DNase-free RNase A (5 µg/mL) (SIGMA), and incubated these at 65°C for 4 h with shaking. After deactivating proteinase K, we removed the beads via magnetic separation to retain the supernatant, which was then subjected to DNA purification using Active Motif’s ChIP DNA Purification Kit.

We analyzed the purified DNA samples using quantitative real-time PCR via the Applied Biosystems Real-Time PCR system (7500 system) and Maxima SYBR Green/Rox qPCR Mastermix (Thermo Fisher Scientific). Primers used included: RET forward (AAGATCCGGCATGTGTGGTT), RET reverse (GCCTTTGGGATCAGTGGACA), CCDC6 forward (GCCACAACACGGTAGAGGAT), CCDC6 reverse (AAGGAAACCTGATGCCCCAC), and GAPDH-1 set (Active Motif).

### BrdU-γH2AX staining

Cells were prepared in 6-well plates at an optimal density and irradiated. Four days post-irradiation, the cells were treated with 10 μM of BrdU (MERK) for 10 min, then collected via trypsinization and fixed with 80% ethanol. After 24 h at -20°C, the fixed cells were denatured with pepsin (HCl 30 mM, Pepsin 0.5 mg/mL) for 20 min at 30°C. This was followed by a 20-min incubation with 2 M HCl at room temperature. Upon pelleting the cells, the resulting cell pellets were re-suspended in a staining solution filled with primary antibodies against γH2AX (Millipore) and BrdU (Dako), diluted in a dilution buffer (FBS 0.5%, Tween 20 0.5%, and HEPES 20 mM), and left in dark for 45 min. After this, cells were washed with PBS and incubated with secondary antibodies in dark for 30 min. After a final washing process, the cells were analyzed using BD AccuriTM C6 (BD Biosciences), and the mean fluorescence of γH2AX in BrdU-positive and BrdU-negative cells was compared.

### Quantification and statistical analysis

We performed statistical analyses with GraphPad Prism software. We used either one- or two-way analysis of variance (ANOVA) or Student’s *t*-test to parse our data. We labeled the results as significant at *P* < 0.05.

## Results

### Induction of DNA damage at post-irradiation

**Figure 1:**
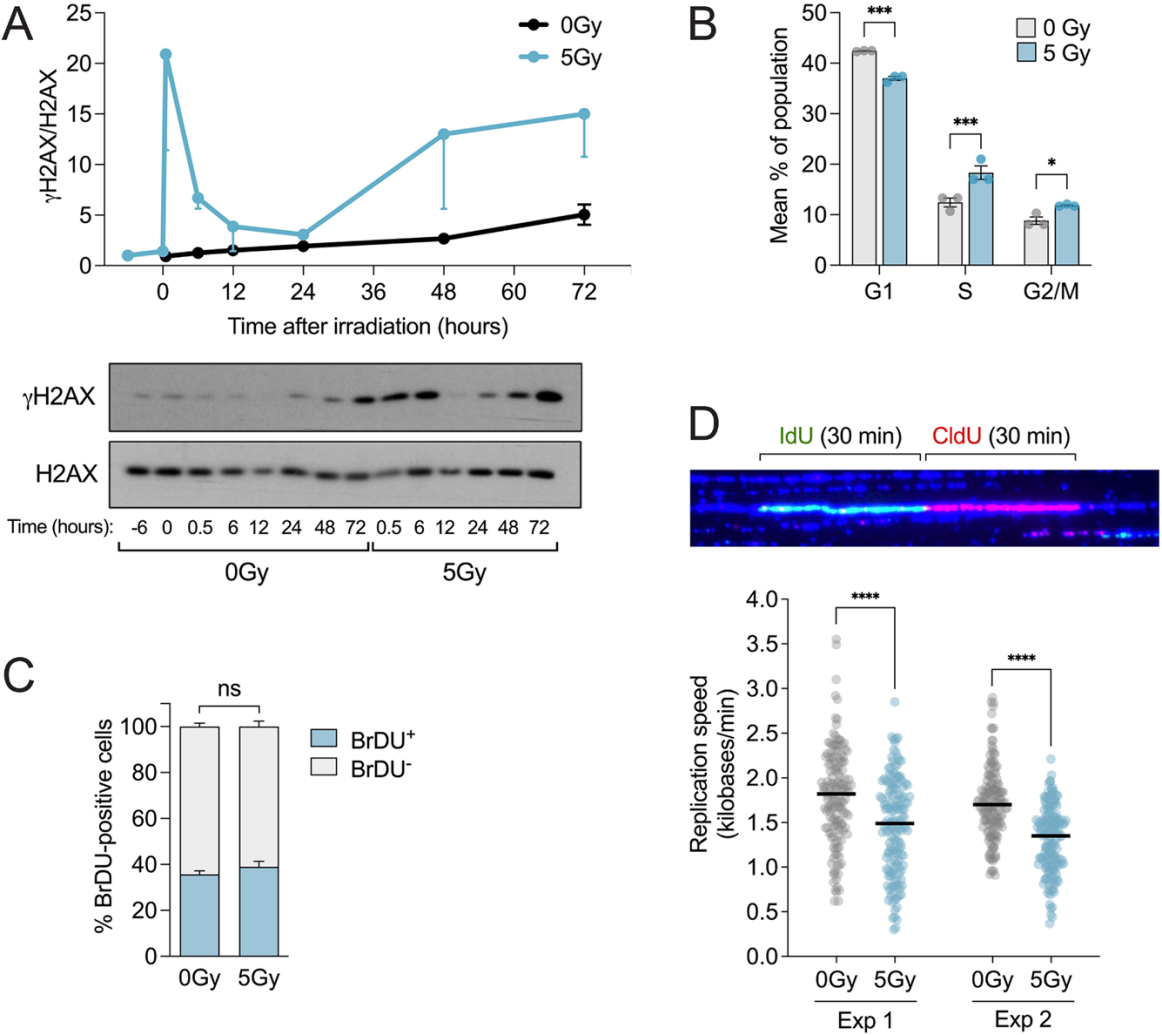
Induction of replicative stress post-irradiation. (A) DNA damage evaluated by phosphorylation of histone H2AX in thyroid cells irradiated or not with 5 Gy using X-rays. γH2AX and total H2AX were detected by Western blotting with specific antibodies. This Western blot is representative of two independent experiments. (B) Cell cycle analysis of non-irradiated and irradiated cells using propidium iodide (*n* = 3) 4 days post-irradiation. (C) Quantification of the proportion of proliferative cells in non-irradiated and irradiated cells by BrdU incorporation (*n* = 3). (D) Measurement of replication fork speed of non-irradiated and irradiated thyroid cells 4 days post-irradiation. Upper panel, an example of a replication fork observed with IdU (green), CldU (red), and anti-DNA (blue) labeling. At least 150 DNA tracks were measured in each condition for every experiment. Black lines represent the mean of fork speed. ****P value < 0.0001, ***P < 0. 001, *P < 0.05.

We studied the time-course levels of total and phosphorylated histone H2AX (Ser139), an established indicator of DNA DSBs, in the non-tumor thyroid cell line (HThy-ori-3.1) post-5 Gy X-ray irradiation (Fig. 1A). Our previous study indicated that this dose triggered the occurrence of RET/PTC1 rearrangement but did not impact the cells’ viability (8). Western blot analysis revealed two damage phases: the first within 12 h, representing irradiation-caused lesions that repair quickly, and the second starting after 24 h and enduring to 72 h (Fig. 1A).

To evaluate the impact of DNA damage following irradiation on cell cycle progression, we executed a propidium iodide (PI)-based cell cycle analysis 4 days post-radiation. Our findings showed that 5 Gy irradiation mildly impacted cell proliferation, with a minor drop in the G1 phase and a small rise in both the S and G2/M phases (Fig. 1B). Analysis of BrdU-positive cell proportions 3 days post-irradiation affirmed that the radiation dose did not significantly affect cell replication (Fig. 1C). However, as the cells tended to accumulate in the S phase, this finding implies the possibility of replicative stress.

We, therefore, examined replication speed using molecular DNA combing at day four post-radiation. Cells were successively incubated with the thymidine analogues 5-iodo-2′-deoxyuridine (IdU) and 5-chloro-2′-deoxyuridine (CldU) to label newly synthesized DNA tracks. Fig. 1D indicates that non-irradiated cells’ fork progresses at nearly 1.82 kb/min (first experiment) and 1.70 kb/min (second experiment), whereas irradiated cells’ fork speed drops to 1.49 kb/min (first experiment) and 1.35 kb/min (second experiment), constituting a moderate speed reduction.

**Figure 2:**
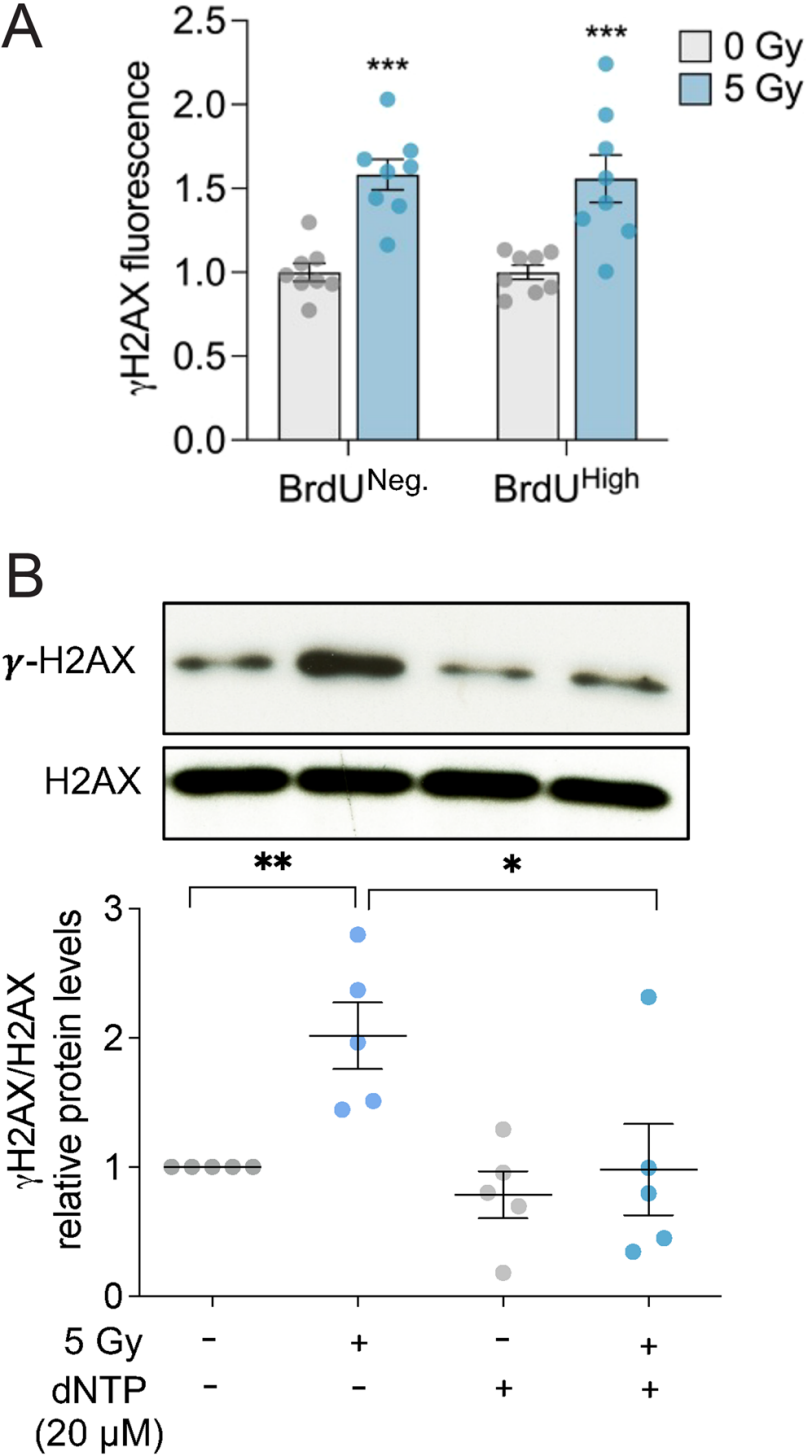
An exogenous supply of dNTP prevents radio-induced DNA damage at day 4 post-irradiation. (A) Detection of DNA damage (γH2AX) in proliferative (BrdU-positive) and non-proliferative (BrdU-negative) cells by flow cytometry in non-irradiated and irradiated thyroid cells 4 days post-irradiation (*n* = 3). (B) The effect of the addition of dNTPs on DNA damage (γH2AX) was analyzed 4 days post-irradiation (5 Gy) in non-irradiated and irradiated HThy-ori-3.1 thyroid cells (*n* = 3). In all experiments, dNTPs were added just before irradiation and 24 h after irradiation during the change of medium. Values are mean ± SE. *P < 0.05; **P < 0.01.

To further verify the continuous proliferation of irradiated cells, we analyzed the levels of γH2AX as a measure of radiation-induced DNA damage in cells labeled with BrdU. Fig. 2A shows that irradiated cells, whether proliferative (BrdU-positive) or non-proliferative (BrdU-negative), exhibit similar levels of γH2AX. This suggests a build-up of persistent DNA damage in G1-phase that does not impede cell cycling. Since nucleotide pool imbalance can induce replication stress (13), we conducted a Western blot analysis to examine the impact of an external supply of dNTP on γH2AX expression post-irradiation. We found that supplementing with nucleotides significantly reduced the induction of γH2AX following irradiation.

### RET is broken at post-irradiation

**Figure 3:**
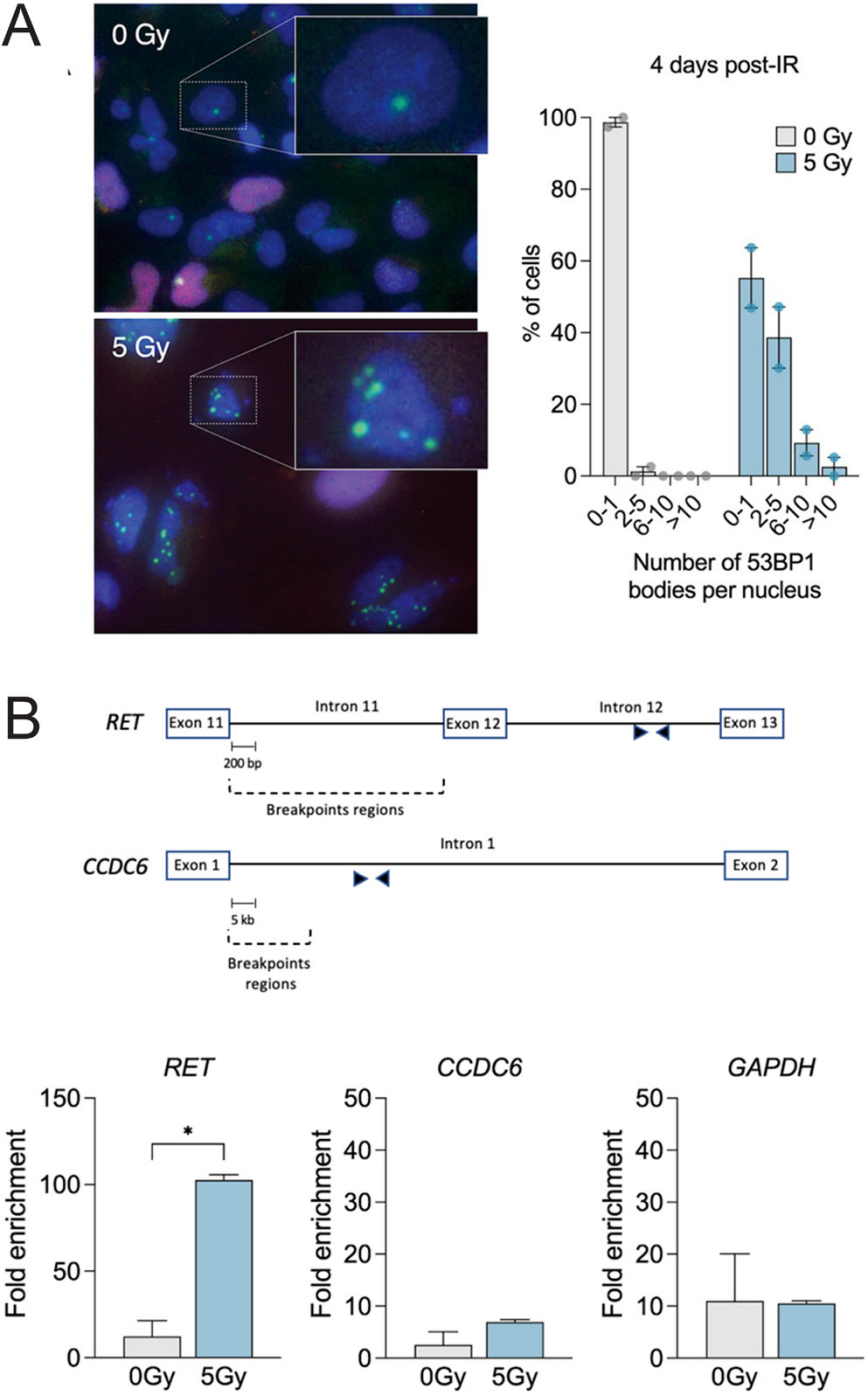
*RET*, but not *CCDC6*, is broken at post-irradiation. (A) Immunofluorescent detection of persistent DNA damage foci in irradiated HThy-ori-3.1 thyroid cells was carried out by probing for 53BP1 (green), cyclin A (red), and DNA (DAPI; blue) at day 4. Right panel: histogram showing the quantification of 53BP1 bodies in G1 nuclei. Cyclin A-negative cells were scored and classified in the indicated categories based on the number of 53BP1 nuclear bodies. The data shown are from two repeats. (B) In this panel, a summary of the breakpoint locations within the *RET* intron 11 (top) and the *CCDC6* intron 1 (bottom) is shown. Filled arrows indicate primers’ locations. At the bottom of the figure, γH2AX level was evaluated at selected genes in untreated and irradiated thyroid cells 4 days after irradiation. The plots represent the enrichment of γH2AX assessed by ChIP-qPCR at indicated gene loci. The data presented are representative of two repeats.

The 53BP1 protein forms larger clusters called 53BP1 nuclear bodies (53BP1-NB) in concert with other signaling elements of DSBs, especially during the G1 phase after replicative stress (14). To verify the lingering under-replicated DNA in mitosis, we examined the formation of 53BP1-NB in G1 daughter cells (cyclin A negative) on the fourth day after irradiation (Fig. 3A). We found an increase in 53BP1-NB numbers in irradiated cells compared to non-irradiated cells. Since the presence of 53BP1-NB at 4 days post-irradiation implies under-replicated DNA, we gauged the occurrence of DNA breakage at both *RET* and *CCDC6* genes, conducting Chromatin immunoprecipitation-quantitative polymerase chain reaction (ChIP-qPCR) analysis with an antibody against γH2AX (Fig. 3B).

The rearrangement involving *RET* and *CCDC6* takes place with a 2 kb intron 11 of *RET* and a substantially large (50–70 kb) intron 1 of *CCDC6* (15). Because the breakpoint locations in *RET* are spread out within intron 11 and the breaks in *CCDC6* intron 1 happen more at the 5′-end (15), these two areas are unsuitable for ChIP-qPCR analysis. However, as γH2AX spreads a limited distance up to 1–2 Mbp from the DNA break site in mammalian cells, we created primers targeting intron 12 of *RET* and a remote area from the breakpoints of *CCDC6* intron 1. Figure 3B demonstrates a γH2AX enrichment in genomic areas within the *RET* gene on the fourth day post-IR, indicating that *RET*, not *CCDC6*, is preferentially broken under this condition.

### The replication rate of RET is delayed at post-irradiation

To execute genome-wide replication-timing profiling, we pulse-labeled HThy-ori-3.1 cells with BrdU and divided them into early and late S-phase fractions using fluorescence-activated cell sorting (FACS) (refer to methods (11)). The freshly synthesized DNA of each fraction was subjected to BrdU immunoprecipitation and specific labeling (Cy3 for the early fraction, Cy5 for the late one) prior to co-hybridization on microarrays. We gauged the replication timing of genomic domains by recording the log2 ratio of early versus late fractions, statistically processed using the START-R software with a *P* value of 0.05 (11). We then contrasted the replication timing in irradiated and non-irradiated HThy-ori-3.1 cells 4 days post-irradiation. START-R analysis discovered that 5.4% of the entire genome was impacted, with 23% (in base pairs) of these regions registering advanced timing and 73% evidencing delays. The findings show that while the *RET* and *CCDC6* genes both go through a late S-phase replication in thyroid cells, irradiation causes an additional *RET* gene replication delay. This finding sheds light on the elevated breakage susceptibility of *RET* following irradiation. Comparing the replication profiles of *RET* and *CCDC6* across various cell types, for which the genomic data of replication timing have been previously determined except for TPC-1 (10), mirrored the patterns encountered in thyroid cells, save for HeLa cells, where an early replication of *CCDC6* was noticed.

## Discussion

Thirteen distinct types of *RET/PTC* rearrangements, each involving a translocation of the *RET* oncogene with a unique partner gene, have been discovered (16). These rearrangements are among the most frequent mutations in papillary thyroid carcinoma (PTC). IR reportedly generates these *RET/PTC* rearrangements, as demonstrated by their high prevalence in radiation-induced PTC (16). IR is known to have delayed cell effects, such as genomic instability, which results in an accumulation of genomic mutations and chromosomal rearrangements. Additionally, radiation-exposed cells face an elevated risk of genomic instability due to lingering replication-stress-associated DSBs caused by the radiation (17). Actively dividing thyroid cells are more at risk from IR than stationary cells (18). Since thyroid cell proliferation is more vigorous in childhood than in adulthood, young thyroids are assumed to be more radiosensitive. The non-cancerous human thyroid cell line HThy-ori-3.1 has proven useful for studying *RET/PTC* rearrangement after *in vitro* radiation exposure (8, 19). Under conditions that permitted the detection of *RET/PTC1* formation 2 weeks after a single 5 Gy X-radiation dose, we observed two waves of DSBs: the first, immediate and resolved within 24 h post-radiation, and the second, delayed, presenting several days after radiation (8). This second γH2AX wave was found in BrdU-positive cells, implying DSB formation during replication. The effects of post-radiation replication stress on chromosome integrity were also evidenced by the presence of 53BP1 nuclear foci in G1-phase cells, indicating that DNA damage penetrates mitosis and affects subsequent generations.

Certain genomic regions are more susceptible to DSBs induced by replication stress. These areas, known as CFSs, are highly responsive to replication stress (20). Two genes, *RET* and *CCDC6*, which are involved in the oncogenic translocation of *RET/PTC1*, inhabit these CFS (4). In thyroid cells, these two gene loci are located in closer spatial proximity than in other tissues, favoring the formation of the *RET/PTC1* translocation in these cells (21).

To the best of our knowledge, this is the first study to illustrate that replication stress, occurring several days post-irradiation, induces breakage in the genomic region of *RET*, but not *CCDC6*, in thyroid cells (Fig. 3). This supports the notion that the fragility of *RET* and *CCDC6* is dependent on distinct sets of conditions that induce fragile sites (4). Specifically, *RET* is prone to substantially higher degrees of chromosomal breakage following chemical treatments that cause disruptions in replication.

**Figure 4:**
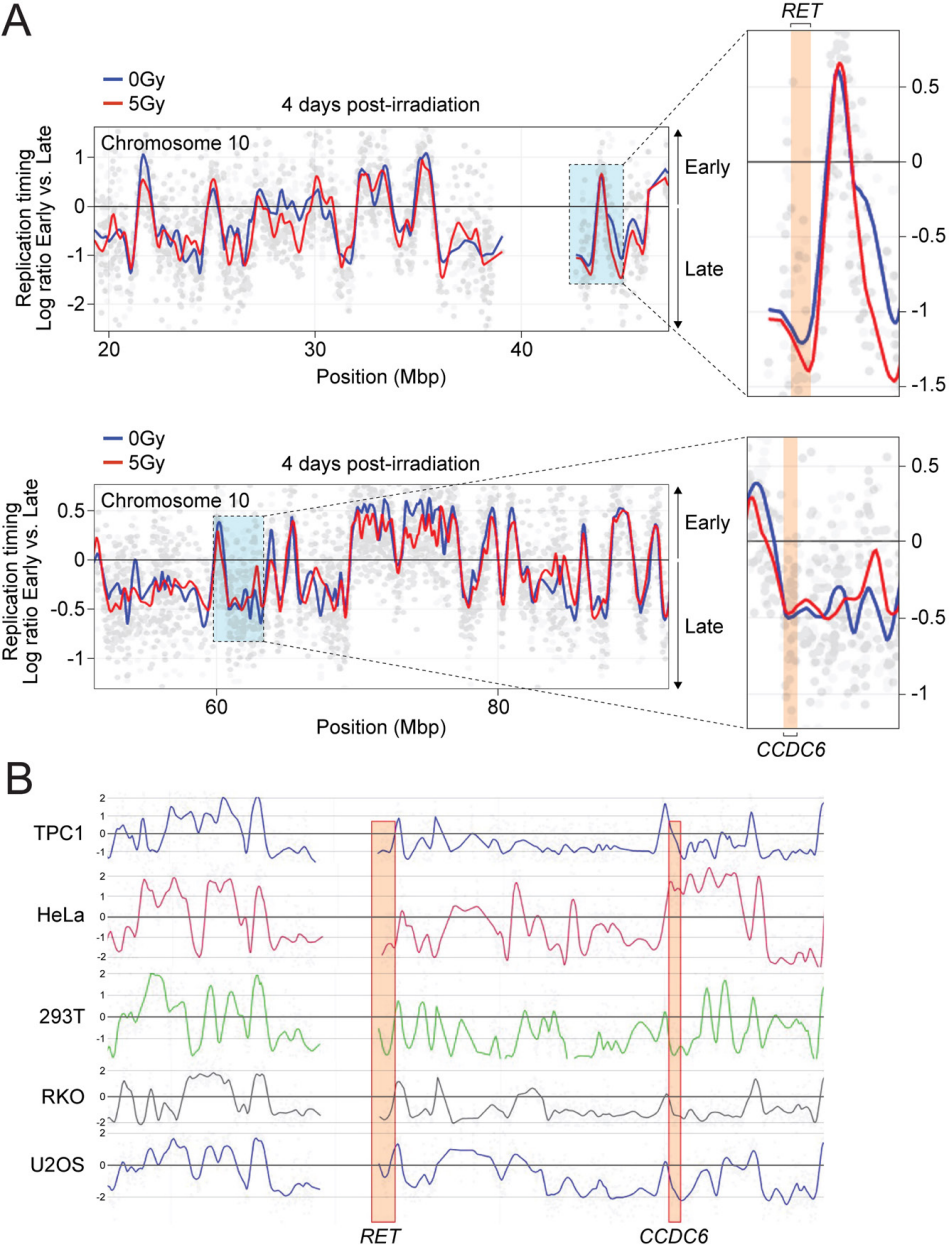
The replication rate of *RET* is delayed post-irradiation. (A) Microarray profiles of the timing of replication on a small section of chromosome 10 from non-irradiated (blue line) and irradiated cells (red line) at 4 days post-irradiation. On the right side, zoomed images show *RET* and *CCDC6* genomic sites (highlighted in orange). Positive values indicate early replication in S-phase and, conversely, negative values indicate late replication in the S-phase. Two replicates were performed. The Student's statistical test was performed directly by the START-R software. (B) Microarray profiles of the timing of replication on chromosome 10 genomic regions containing *RET* and *CCDC6* from non-irradiated TPC1, HeLa, HEK293T, RKO, and U2OS cell lines. *RET* and *CCDC6* genomic sites are highlighted in orange. The replication timing data come from the analysis of genomic data of replication timing in the five human model cell lines previously published (10).

The instability of CFSs upon replication stress could be explored by understanding their replication dynamics, including replication timing. For instance, *RET* and *CCDC6*, which both replicate late in the S-phase (Fig. 4) – a shared trait among CFSs (22) – can illustrate this. Yet, following irradiation, the replication timings of *RET* and *CCDC6* are affected differently. While *CCDC6*’s replication timing remains consistent, *RET*’s replication is further delayed post-irradiation.

CFSs are predominantly situated in large transcribed domains with a scarcity of initiation events (7). The combination of CFSs’ late replication timing and replication scheme via long-traveling forks often leads to incomplete replication near the mitotic entry. Therefore, any delay in *RET* replication in the late S-phase post-irradiation could heighten the possibility of replication failure, which may account for why *RET* is more likely to break post-irradiation.

Nevertheless, it is essential to note that *RET* also undergoes late replication in cells derived from tissues where *RET/PTC* translocations are uncommon. This observation emphasizes that the specific spatial proximity between *RET* and its partner genes in the thyroid may be a critical factor in *RET/PTC* translocations in these cells.

We found that providing an external supply of nucleosides can minimize DNA damage after IR (post-IR). Both the balance and overall concentrations of dNTPs are essential for precise DNA replication. Since dNTP concentrations remain stable in mammalian cells after DNA damage due to irradiation (23), it is plausible that a decrease in dNTP availability leads to a slowdown in replication forks. Furthermore, the accumulation of endogenous damage may cause dNTPs to be used for DNA repair instead of DNA replication, leading to a dNTP shortage for replication and, in turn, replication stress (24).

DNA DSBs can initiate genomic rearrangements through various mechanisms: end-joining (canonical non-homologous end-joining (C-NHEJ) and alternative end-joining (A-EJ)), homologous recombination, microhomology-mediated template switching (MMTS), microhomology-mediated break-induced replication, and fork stalling template switching (25). Whereas C-NHEJ and A-EJ necessitate two DNA double-strand ends, the other mechanisms need only one DSB, capable of invading and copying an unscathed DNA partner. Our findings imply that such mechanisms may be at play in thyroid cells. Consequently, if *RET* experiences a break following replicative stress, it could invade *CCDC6* or other nearby gene partners, which may explain the high prevalence of *RET/PTC* translocations in patients exposed to IR.

Indeed, many thyroid tumors exhibit *RET/PTC1* rearrangements, even without a history of radiation exposure. Our previous study has demonstrated that H_2_O_2_ can cause the *RET/PTC1* rearrangement in thyroid cells. This suggests that oxidative stress alone may be enough to trigger the *RET/PTC* rearrangement (8). Oxidative stress can slow down the replication fork’s speed, leading to replication stress. This can be specifically caused by mechanisms such as oxidative DNA lesions, nucleotide pool imbalances, and replicative DNA polymerase impairments (26, 27). Increasing evidence indicates that replication and oxidative stress are interconnected, mutually enhancing their contributions to genomic instability. We have previously found that the induction of dual oxidase 1 (DUOX1)-derived H_2_O_2_ delays DNA breakage after thyroid cells are irradiated, a factor associated with changes in the nuclear redox environment (28). It would be especially interesting to explore further the role of DUOX1-dependent H_2_O_2_ production in *RET* breakage by examining how it affects replication stress post-radiation exposure in thyroid cells.

## Conclusion

Our study indicates that replication stress in thyroid cells, which occurs several days after a single irradiation event, causes a delay in gene-level replication. This delay results in the formation of DSBs in the *RET* gene, creating ideal conditions for the replicative failure that results in the chromosomal translocation known as *RET/PTC*. The associated risk of radiation in the thyroid could be ascribed to the accumulation of DSBs associated with replication stress rather than directly to DNA breaks caused by radiation – these are typically repairable within a few hours.

## Declaration of interest

The authors declare that there is no conflict of interest that could be perceived as prejudicing the impartiality of the research reported.

## Funding

C Dupuy received financial support from Electricité de France (EDF) and the Institut National Du Cancerhttp://dx.doi.org/10.13039/501100006364 (INCA) CANCEROPOLE-2013-PL BIO-14-CNRS. F Hecht was the recipient of a fellowship from Conselho Nacional de Desenvolvimento Científico e Tecnológico (CNPq, Brazil), and C F Lima-Gonçalves was the recipient of a fellowship from CAPES (Brazil)-COFECUB (France). L Valério was the recipient of a fellowship from the European Thyroid Association. M Harinquet, R A El Hassani, D P Carvalho, S Koundrioukoff, and J-C Cadoret have nothing to declare.

## Author contribution statement

FH performed experiments for time-course analysis of γH2AX expression, FACS analysis, and ChIP-qPCR. LV carried out experiments with DNA combing and immunofluorescence. CFLG performed Western blot analyses. MH performed the immunofluorescence analyses. RAEH and DPC helped design cellular studies. SK helped design experiments with DNA combing. J-CC designed experiments for replication timing and performed genome-wide analysis. CD wrote the manuscript, and all authors reviewed it. CD conceived and planned the study.
